# Torsin A Localization in the Mouse Cerebellar Synaptic Circuitry

**DOI:** 10.1371/journal.pone.0068063

**Published:** 2013-06-19

**Authors:** Francesca Puglisi, Valentina Vanni, Giulia Ponterio, Annalisa Tassone, Giuseppe Sciamanna, Paola Bonsi, Antonio Pisani, Georgia Mandolesi

**Affiliations:** Department of Systems Medicine, University of Rome Tor Vergata/Laboratory of Neurophysiology and Synaptic Plasticity, Fondazione Santa Lucia, Rome, Italy; University of Iowa Carver College of Medicine, United States of America

## Abstract

Torsin A (TA) is a ubiquitous protein belonging to the superfamily of proteins called “ATPases associated with a variety of cellular activities” (AAA^+^ ATPase). To date, a great deal of attention has been focused on neuronal TA since its mutant form causes early-onset (DYT1) torsion dystonia, an inherited movement disorder characterized by sustained muscle contractions and abnormal postures. Interestingly, it has been proposed that TA, by interacting with the cytoskeletal network, may contribute to the control of neurite outgrowth and/or by acting as a chaperone at synapses could affect synaptic vesicle turnover and neurotransmitter release. Accordingly, both its peculiar developmental expression in striatum and cerebellum and evidence from DYT1 knock-in mice suggest that TA may influence dendritic arborization and synaptogenesis in the brain. Therefore, to better understand TA function a detailed description of its localization at synaptic level is required. Here, we characterized by means of rigorous quantitative confocal analysis TA distribution in the mouse cerebellum at postnatal day 14 (P14), when both cerebellar synaptogenesis and TA expression peak. We observed that the protein is broadly distributed both in cerebellar cortex and in the deep cerebellar nuclei (DCN). Of note, Purkinje cells (PC) express high levels of TA also in the spines and axonal terminals. In addition, abundant expression of the protein was found in the main GABA-ergic and glutamatergic inputs of the cerebellar cortex. Finally, TA was observed also in glial cells, a cellular population little explored so far. These results extend our knowledge on TA synaptic localization providing a clue to its potential role in synaptic development.

## Introduction

TA is a member of protein family AAA^+^ ATPase, ubiquitary proteins which typically have a chaperone-like function [1,2]. By acting as homo-oligomeric complexes, these ATPases mediate ATP-dependent conformational remodeling reactions involving substrate proteins or protein complexes [3]. A deletion of a single glutamic acid residue near the carboxy terminus of the protein TA [1] causes DYT1 dystonia, an early-onset severe autosomal-dominant movement disorder with reduced penetrance. Although there is no evidence of neurodegeneration in DYT1 dystonia, the cascade of events which are triggered by the mutant TA and lead to dystonic movements is still unknown. Recent evidence from both clinical studies and animal models point to a concomitant involvement of two sensorimotor regions, basal ganglia and cerebellar pathways [4–6], thus supporting the hypothesis that dystonia may represent a neurodevelopmental circuit disorder [7].

TA is typically localized in the endoplasmic reticulum (ER) and in the inner nuclear membrane and thought to be retained there by association with membrane spanning proteins [8–10]. In neuronal cell culture, TA was found also throughout the cytoplasm, neurite processes, and growth cones [9–13]. By interacting with the cytoskeletal network it can play important roles for neuronal maturation such as cell adhesion, neurite extension, and cytoskeletal dynamics [9–14]. Recent evidence highlights a new function for TA in endoplasmic reticulum-associated degradation (ERAD) in response to stress [15]. Interestingly, it plays also a regulatory role in the secretory pathway [16–18], in the synaptic vesicle (SV) transport [13], turnover [19], and transmitter release [20–23]. Accordingly, TA has been found enriched in synaptosomal membrane fractions at ultrastructural level within axons and presynaptic terminals [24]. Despite such evidence, a detailed analysis of the cellular and synaptic distribution of TA is still lacking. Immunolocalization of TA in adult human, rat and mouse cerebellum [25–30] showed an abundant expression of the protein in PC soma and proximal dendrite, in dispersed cells in molecular layer (ML) and in the DCN. Of note, the protein reaches the peak of expression in rodent cerebellum [30,31] around postnatal day 14 (P14), virtually corresponding to childhood in humans, a period when cerebellar neurons undergo extensive axonal outgrowth, dendritic branching, and synaptic remodelling [32,33]. To better clarify the potential role of TA in the cerebellar neurodevelopment, we characterized its expression profile in the cerebellum of normal juvenile mice. In addition, since scarce information is available on TA localization at synaptic terminals, we performed by a quantitative confocal analysis a detailed study of its expression within the main GABA-ergic and glutamatergic inputs of the cerebellar circuitry.

## Experimental Procedures

### *Mice*

C57BL/6 mice (The Jackson Laboratory, Bar Harbor, ME, USA) were used for the experiments. Experimental procedures and handling of mice were carried out in strict accordance with the EC and Italian guidelines (86/609/EEC; D. Lvo 116/1992) and approved by the Santa Lucia Foundation Animal Care and Use committee (protocol 119/2009-B and n. 07/2011). All the efforts were made to minimize the number of animals utilized and their suffering.

### *Western blot*

Cerebella were obtained from anesthetized mice at different age: P7, P14, P21, P60. Cerebella were homogenized with cold RIPA buffer (50 mM Tris pH 7.5, 300 mM NaCl, 1% Triton X-100, 10% glycerol, 1.5 MgCl_2_, 1 mM CaCl_2_, 1 mM ethylene glycol tetraacetic acid, and 1% protease inhibitor cocktail, Sigma) sonicated and left in ice for 1 hour (h); then crude lysates were centrifuged at 13000 rpm for 15 min at 4°C, the supernatant collected and quantified with Bradford assay. Protein extracts were stored at -80°C. 5 µg of each sample in Page LDS sample buffer 4x (Invitrogen) was analyzed to detect TA variation of expression during development; samples were denatured at 98°C for 5’ and were loaded onto the sodium dodecyl sulfate (SDS) polyacrylamide gel 12% (Biorad). Gels were blotted onto a 0.45 µm polyvinylidene fluoride (PVDF) membrane. Immunodetection was performed by rabbit anti-TA 1:500 (AbCam cat. num. ab34540) and mouse anti-β actin (Sigma, A5441) was used as loading control. Horseradish peroxidase (HRP)-conjugated secondary antibodies (Bio-Rad), ECL-Plus reagent (GE Healthcare, Europe) and the storm 840 acquisition system were used for detection of the proteins of interest. Quantification of the bands intensity on scanned filters was achieved by ImageJ software [34]. The experiments were performed in triplicates, data form each experiments were normalized to adult age values and pulled together for statistical analysis. Data were statistically compared using the One-way ANOVA post hoc Tukey’s test. Differences were considered significant if p< 0.001. The results were presented as means ± SEM.

### *Synaptosomal preparation*

Samples were processed according to the synaptosomal preparation previously described [35]. Briefly, P14 cerebella were homogenized through Dounce homogenizer in 2 ml of the homogenization buffer (0.32M sucrose, 1mM EDTA, 1mg/ml BSA, 5mM HEPES at pH 7.4) with Na _3_VO_2_ (25mM), protease inhibitor cocktail (PIC, 1% Sigma cod P8340) and NaF (10 mM). The crude homogenates were centrifuged at 3000 x g for 10 min at 4°C; subsequently the supernatants were centrifuged at 14000 x g for 12 min at 4°C and the resultant pellets were resuspended in 220µl in Krebs-Ringer buffer (140mM NaCl, 5mM KCl 10 mM glucose, 25 mM HEPES at pH 7.4 plus 1% PIC) and 180 µl of Percoll (Sigma-Aldrich cod. P7828). Samples were then centrifuged for 2 min at 14000 x g. The synaptosome fraction of the gradient was removed, washed more times with Krebs–Ringer buffer by centrifugation for 1 min at 14000x g. The final pellets were resuspended in 40 µl of Krebs–Ringer buffer. All steps of the procedure were performed at 4°C using ice-cold buffers. Each sample was resuspended in 1% SDS, boiled and sonicated. The protein content was quantified and aliquots were frozen at -80°C until use. The protein extracts (20 µg) were loaded onto 12% polyacrylamide gel and transferred onto PVDF membranes. The blots were probed using: rabbit anti-TA antibody 1:500 for 3 over nights (ON) (AbCam, cod. 34540), rabbit anti-vesicular glutamate transporter 1 (V1) 1:15000 for 1 ON (SY–SY, cat. num. 135302), rabbit anti-vesicular glutamate transporter 2 (V2) 1:5000 for 1 ON (SY–SY cat num 135103); rabbit anti-vesicular GABA transporter (VGAT) 1:1000 for 1 ON (SY–SY, cat. num. 131011), rabbit anti-Synapsin-I 1:1000 for 1 ON (Novus Biologicals NB300-104), mouse anti-post synaptic density 95 protein (PSD95) 1:20000 1h (Millipore, cod. MAB1598) and mouse anti-beta actin 1:10000 for 30 min (Sigma, A5441). HRP-conjugated secondary antibodies (Bio-Rad), ECL-Plus reagent (GE Healthcare, Europe GmbH), and the Storm 840 acquisition system were used for detection. All the experiments were performed in triplicates.

### *Immunohistochemistry*

Mice were deeply anesthetized and whole body perfused with cold 4% paraformaldehyde in 0.12 M phosphate buffer at pH 7.4. Cerebellum was dissected, post fixed for at least 3h at 4°C and equilibrated with 30% sucrose ON. Sagittal cerebellar sections (30 µm thick) were cut with a freezing microtome and put in PBS. The slices were first dehydratated with serial alcohol dilutions (50-70-50%) to improve the antigen retrieval and lower the background [36] and then were blocked 1h at RT with 10% donkey serum solution (NDS) in PBS 0.25% Triton X-100 (PBS-Tx). Slices were incubated with the primary antibodies for 3 days in mild agitation at 4°C, with the following dilution: mouse monoclonal anti-Calbindin D-28K (Cb) 1:3000 (Swant, cat. num. 300); rabbit polyclonal anti-TA 1:600 (Abcam, cat. num. ab34540); mouse monoclonal anti-parvalbumin (PV) 1:500 (Sigma-aldrich, cat. num. P3088); mouse monoclonal anti-Glial Fibrillary Acid Protein (GFAP) 1:300 (Immunological sciences, MAB12029); mouse monoclonal anti-Neurofilament H non-phosphorylated (SMI32) 1:500 (Covance, cat. num. SMI-32R); guinea-pig polyclonal anti-V1 1:1500 (SY–SY, cat. num. 135304); mouse monoclonal anti-V2 1:500 (Millipore, cat. num. MAB5504); mouse monoclonal 1:500 (SY–SY, cat. num. 131002) and rabbit polyclonal anti-VGAT 1:500 (SY–SY, cat. num. 131011). After being washed three times with PBS-Tx, the section were incubated with the appropriate mix of secondary antibodies: alexa488 (ms; Invitrogen), alexa 647 (guinea pig; Invitrogen), cyanine 3 (cy3; Rb) and cyanine 5 (cy5; mouse or guinea pig) conjugated secondary antibodies (Jackson ImmunoResearch, West Grove, PA, USA) 1:200 for 2 h at RT in mild agitation and rinsed in PBS-Tx. Section were then washed in PBS-Tx and eventually a Dapi staining (1 µl/ml; Sigma Aldrich, cat. num. D9564) was performed in PBS-Tx for 10’ at RT. Sections were then washed twice and mounted with Vectashield® mounting medium on plus polarized glass slides (Super Frost Plus Thermo Scientific) and coverslipped. To test the specificity of primary antibody we follow the same procedure with the exception that the antibody anti TA was preincubated for 1 h with an excess of the specific TA peptide (PepTA) of 1:2,5 (Abcam, cat. num. ab35510). To test the specificity of the secondary antibody, the slices were incubated without the primary antibody anti-TA and then normally processed. No signal was detected, confirming further the specificity of the TA immunodetection.

### *Confocal imaging*

All images were acquired with a LSM700 Zeiss confocal laser scanning microscope (Zeiss, Göttingen, Germany) and processed using NIH ImageJ software [34]. A 10x objective was used for acquisition at low magnification. A 63x/NA1.4 oil objective was used for acquiring all the other images, to clearly resolve synaptic contacts an additional digital zoom factor of 1,5x was used. A 2,5x zoom was specifically applied for Purkinje Cells-Parallel Fibers (PC-PF) synaptic contacts acquisitions. Section images (1024 x 1024) in the z-dimension (z-spacing, 0.3 µm) were collected, ensuring that an entire segment of a dendrite/synaptic contact, spanning multiple confocal planes, was fully captured from the surface of the section. Z stacks images were acquired to better identify synaptic contacts. The confocal pinhole was kept at 1, the gain and the offset were adjusted to prevent saturation of the brightest signals and sequential scanning for each channel was performed. The confocal settings, including laser power, photomultiplicator gain and offset were kept constant for each marker. Accordingly, the immunostaining procedures on the slices used for quantitative analysis were processed all together. For each quantitative analysis we collected images from 4–5 slices of each cerebellum (n = 3). In particular, in each slice we acquired at least 2 images in the same lobules (V–VI). Confocal images were exported in TIFF file format and adjusted for reducing noise by applying background subtraction as required by the NIH ImageJ software. To better visualize the colocalization signal between TA and specific markers we created a colocalization mask (white) by using ImageJ. To this aim an intensity threshold for each channel after acquisition was applied. The mask exclusively visualizes the overlapping pixels of the two channels.

### *Quantitative colocalization analysis*

The quantitative colocalization analysis was performed by means of the ImageJ software, in particular by using the Just Another Colocalization Plugin (JACoP) plugin [37] and the Intensity Correlation Analysis (ICA) plugin (WCIF ImageJ) as described by Li et al. (2004) [38]. Both analysis were conducted on synaptic structures in which both pre- and post-synaptic sites were clearly defined not only in the x-y dimension but also in the z-axis. To this aim we selected the best optical section in the z-stack in terms of intensity signal and area occupied by the synaptic structures.

We first isolated sets of synaptic contacts in a single optical section and calculated the overlap coefficient r as reported by JACoP: ( ∑i (Ai x Bi ))/√(∑i (Ai)2 x ∑i(Bi)2) [39]; where Ai and Bi represent signal intensities of pixels for each channel. This coefficient varies from zero to one, the former corresponding to non-overlapping signals and the latter reflecting 100% of colocalization between both signals. The total number of synaptic contacts was reported for each group of synapse in the results.

The ICA was performed on isolated single synaptic structures along the z-stack (0.3 µm step). To this aim, besides analyzing the best central section (as described above) we analyzed also the up and down sections. This type of analysis generates scatter plots (ICA plot) of stain A or stain B against the product of the differences from the mean (PDM) which is the product of the difference of each pixel A and B intensities from their respective means as described in Li et al. 2004 [38]. The resulting plots emphasize the high intensity stained pixels and allow the identification of protein pairs that vary in synchrony (positive PDM values), randomly (around 0), or independently (negative PDM values) within the cell. The ICQ value is based on the non-parametric sign-test analysis of the PDM values and is equal to the ratio of the number of positive PDM values to the total number of pixel values. The ICQ values are distributed between -0.5 and +0.5 by subtracting 0.5 from this ratio. Random staining: ICQ~0; Segregated staining: 0> ICQ > -0.5; Dependent staining: 0<ICQ <+0.5.

#### Negative controls

to assess the reliability of both methods, we calculated for each set of selected synaptic structures the overlapping coefficients r and the ICQ values originating from two markers which are known to be segregated in the pre- and post synaptic sites (negative control, CTR). In details in the ML we analyzed: Cb-positive spine/V1-positive terminals (Cb/V1) as CTRs of both PC-spines and PFs; Cb-positive PC dendrites/V2-positive terminals (Cb/V2) as CTR of Climbing Fibers (CFs), Cb-positive PC body/VGAT-positive terminals (Cb/VGAT) as CTR for GABA-ergic terminals (we exclude the contacts with a strong colocalization Cb-VGAT due to the presence of VGAT positive/Cb-positive PC collaterals on PC bodies). In the DCN, SMI32-positive DCN body/VGAT-positive terminals (SMI32-VGAT) as CTR for PC synaptic terminals. Regarding to the internal granular layer (IGL), the negative CTR was represented by two presynaptic terminals which are very close to each other in the cerebellar glomeruli, the mossy fibers (MFs) and Golgi synaptic terminals which are positive for V1 or V2 and VGAT (V1/VGAT and V2/VGAT), respectively. Finally, the negative CTRs for GFAP-positive glia cells were represented by very close structures such as in the ML Cb-positive PC dendrites/GFAP processes (Cb/GFAP) and in the IGL by Cb-positive PC axon/GFAP processes (Cb/GFAP).

Statistical analysis: numerical data are presented as means ± SEM. Student’s t test (t test, p) or the normal approximation of the sign test (sign test, p) were used as tests for significance. One-way ANOVA test with a post hoc Holm–Sidak test was performed among groups. The significance level was set at p < 0.05.

## Results

### *Time course of TA expression in the mouse cerebellum*

**Figure 1:**
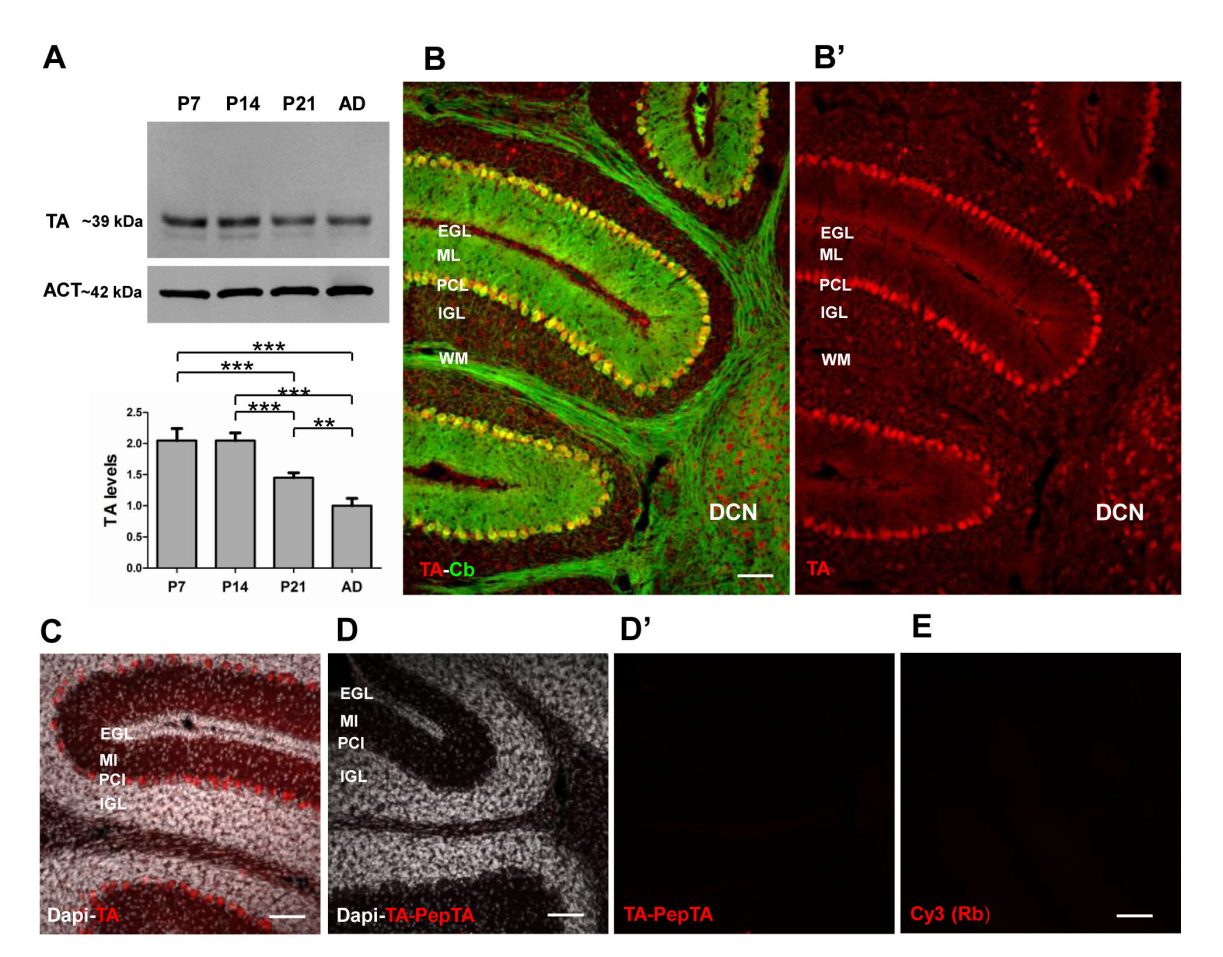
TA expression in the mouse cerebellum. **A**) Western blot of TA protein at various postnatal stages (P7, P14, P21, P60). The amount of TA was quantified relative to actin (ACT). The graph shows the quantitative analysis of TA levels at the various ages normalized to P60. TA was highly expressed during postnatal development (P7-P14) and significantly decreased in the adult age. **B**–**B**’) Double immunofluorescence of a cerebellar sagittal slice at P14 showing the distribution of TA immunoreactivity (red) and Cb which labels PCs (green). **B’** shows a diffuse distribution of TA immunoreactivity in all cerebellar layers (EGL, ML, PCL, IGL and WM) and also in the DCN. **C**–**E**) Specificity of the rabbit polyclonal anti-TA antibody by immunostaining. Serial cerebellar sections incubated either with the anti-TA antibody (red, C) or with the anti-TA antibody preincubated with the TA-antigen specific peptide (**D**–**D**’). Slices were counterstained with Dapi (white). Some slices were incubated only with the cy3-coniugated anti-rabbit secondary antibody (**E**). The absence of signal in D’ and E demonstrated the specificity of the anti-TA antibody. All scale bars: 100 µm. One-way ANOVA in **A**: ** p < 0.01 and *** p < 0.001.

We first performed a time course analysis of TA expression in the mouse cerebellum by western blot. Mice were sacrificed at different ages; P7, P14, P21 and adult (P60). As shown in Figure 1A, TA was highly expressed during the postnatal development at P7 (2.05 ± 0.19; n = 3) and P14 (2.05 ± 0.12; n = 3). At P21 (1.45 ± 0.08, n = 3) the signal decreased to reach half values in the adult age (1 ± 0.12, n = 3) (one-way ANOVA, p < 0.001 and post-hoc Tukey’s test p < 0.001, P7 and P14 vs P21 and P60, p < 0.01 P21 vs P60).

Our goal was to provide a detailed analysis of TA localization in the mouse cerebellum both at cellular and synaptic level. Therefore, we performed immunofluorescence experiments and confocal analysis at P14 when TA reaches high level of expression concomitantly with cerebellar synaptogenesis [33]. In mouse cerebellar sagittal sections we observed a broad expression of the protein throughout the cerebellar cortex (Figure 1B–B’). Observation at a low magnification revealed that the most intense TA immunoreactivity was detected in the Purkinje cell layer (PCL), in the external granular layer (EGL) and also in the DCN. In the ML, besides the presence of PC dendrites intensively stained, numerous marked puncta formed an homogeneous band. Within the internal granule cell layer (IGL) immunolabeling appeared less dense and in a patchy punctuate fashion, in addition sparse cell bodies were intensively stained. TA immunoreactivity was observed also in the white matter (WM).

In order to assess the specificity of the antibody anti-TA, we performed the immunostaining assay of cerebellar slices with the TA antibody previously blocked by the TA immunizing peptide (Figure 1D–D’). As shown in Figure 1D’ the TA signal disappeared in the slices, confirming the specificity of the TA staining. To exclude aspecific signal derived from the secondary antibody we performed also an immunostaining of slices incubated only with the secondary antibody. Accordingly, as shown in Figure 1E no signal was detected. The specificity of the antibody was further confirmed by western blot analysis in cerebellar lysates (Figure 1A), where one intense band was detected approximately at 39 kDa likely corresponding to the molecular weight of the glycosylated TA form, while the lower band likely represents the native protein [24].

### *TA expression in the different cellular populations of the cerebellar cortex at P14*

**Figure 2:**
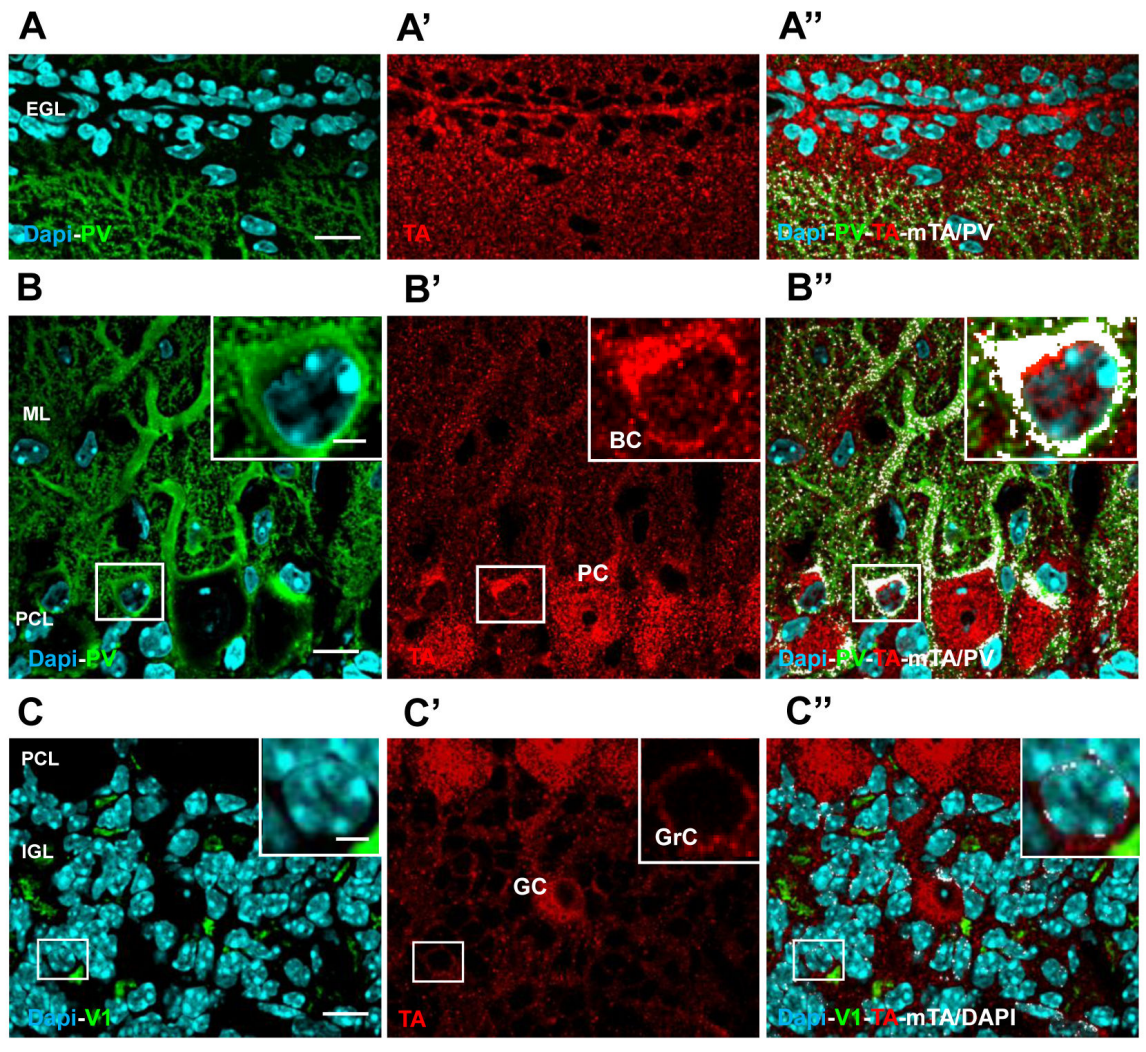
TA localization in the mouse cerebellar cortex at P14. **A-A**’’and **B–B**’**’)** Cerebellar sagittal section immunostained for PV (green) and TA (red) and counterstained with Dapi (cyan). **A**) Merge image between Dapi-positive nuclei (cyan) and PV-positive PC dendrites (green) in the upper part of the ML and the EGL. **A**’) Shows the corresponding TA immunostaining (red). **A**’’**)** Merge image of all markers (PV-TA-Dapi) plus the colocalization mask TA/PV (mTA/PV in white) highlighting the expression of TA in PC dendrites and spines. **B**) Merge image of PV staining and Dapi-positive nuclei, highlighting the PV-positive PC bodies and dendrites and interneurons of the ML (green). The inset (white box) is an high magnification of a BC body. **B**’) TA protein (red) is abundantly present in PCs and BCs (inset), both in the cytoplasm and nuclei. Black spaces are TA-negative nuclei in the ML. **B**’’) merge image of all markers (PV-TA-Dapi) plus the colocalization mask TA/PV (mTA/PV in white). **C**) Merge image between DAPI-positive nuclei and V1-positive (green) rosettes to define the IGL. Most of the DAPI-positive nuclei in the IGL belong to granule cells **C**’) TA staining is diffuse also in this layer; granule cells (high magnification in the inset) and Golgi-like cells are intensively labeled. **C**’’) Merge image of all markers (TA-V1-Dapi) plus the colocalization mask TA/Dapi (mTA/Dapi in white). The absence of the white mask highlights no expression of TA in almost all cell nuclei. Few white dots in the inset indicate overlapping signal of TA around the nucleus. Scale bars in A, B and C 10 µm; in the insets 2 µm.

**Figure 3:**
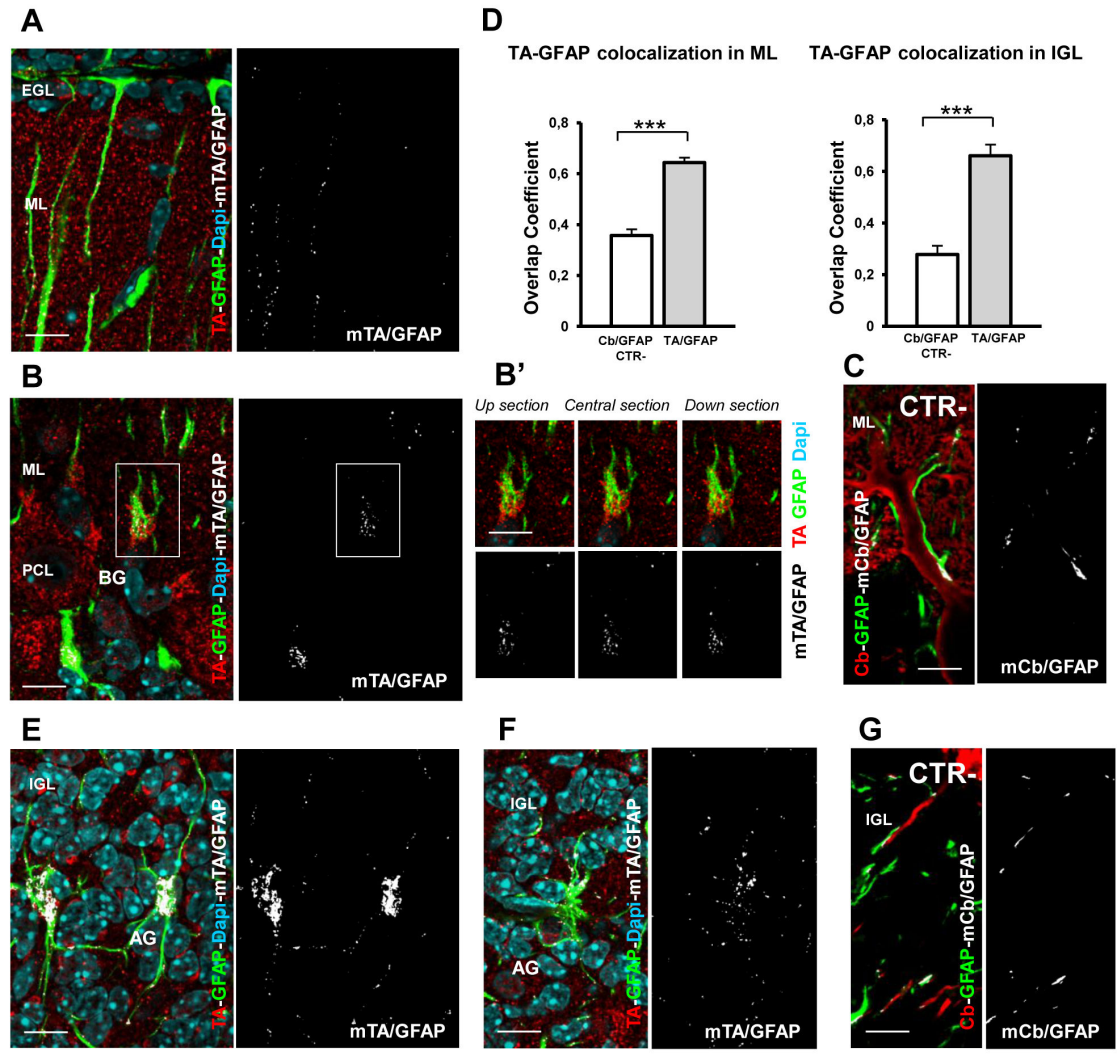
TA expression in glial cells of the cerebellar cortex at P14. **A**, **B**, **E**, **F**) Merge images between DAPI-positive nuclei (cyan), TA immunostaining (red) and GFAP-positive glia cells (green) in cerebellar sagittal slices. **A**) The colocalization mask TA/GFAP (mTA/GFAP, white) highlights the expression of TA in Bergmann glia (BG) palisades and endfeets in the upper ML and EGL. **B**) The colocalization mask TA/GFAP (mTA/GFAP) shows TA localization in glia cells localized in the PCL and ML. **B**’) Serial sections of the inset in B highlights TA expression (red) inside the cell body of BG. **C**) Merge between Cb-positive PC dendrites (red), GFAP and the colocalization mask Cb-GFAP (mCb/GFAP) in the ML. The overlapping signal between these two markers represent the negative control for quantification analysis. **D**) Quantitative colocalization analysis of TA expressed in GFAP-positive glia cells in the ML (left panel) and IGL (right panel). The mean values of the r overlap coefficients of TA/GFAP (gray columns) were significantly different from the negative CTR Cb/GFAP (white column), indicating TA localization in glia cells. **E**–**F**) The colocalization masks TA/GFAP (mTA/GFAP) in E and F show a differential expression of TA in bushy glia cells localized in the IGL. **G**) Merge of Cb-positive PC axon (red), GFAP (green) and the colocalization mask Cb-GFAP (mCb/GFAP) in the IGL. The overlapping signal between these two markers represent the negative control for quantification analysis. *** p < 0.001. Data are represented ad mean ± SEM. Scale bars, 10 µm.

The four layers of the juvenile cerebellar cortex (ML, PCL, EGL and IGL) were easily recognized in sagittal cerebellar sections by using the nuclear marker (Dapi) which labels almost all the cellular nuclei (Figures 2 and 3). In addition, to better define the expression of TA in different cell types, we used specific markers in combination with topological and morphological characteristics. Finally, we report the colocalization signal (mask) between TA and the specific marker to highlight the expression of the protein in the different neuronal populations of the cerebellar cortex.

#### Purkinje cell layer and molecular layer

PCs, which were labeled with an antibody anti-PV (green, Figure 2A-B), have their large soma organized in a monolayer (PCL), while the dendrites extend densely through the ML toward the boundary of the cortex (green, Figure 2A-B). TA (red, Figure 2B’) was abundantly expressed in the soma and dendrites of PCs as highlighted by the colocalization mask (mTA/PV, Figure 2B’’). At P14 the PC dendritic arbor is still immature in particular in the upper part of the ML (Figure 2A); this layer expands during the development until P21, as a result of the inside-out stacking of newly-forming parallel fibers (PFs), which are the axon of granule cells located in the IGL (Figure 2C). During development granule cells originate in the EGL above the ML (Dapi-positive cells, Figure 2A) and migrate through the ML (Figure 2B) toward the IGL (Figure 2C). Also basket (BC) and stellate cells originate and migrate from the EGL by P14 to the ML. These cells are GABA-ergic interneurons of the ML exerting an inhibitory effect on PCs and are typically labeled with antibody anti-PV (Figure 2B). As shown in Figure 2B’’, TA (red) was localized also in the soma of PV-positive neurons which are likely BCs since at this time these cells are already localized in the lower part of the ML. We could not distinguish stellate cells since they appear during the second week of postnatal life and mature over an extended period.

#### External and internal granular layer

A significant TA signal was present also in the EGL (Figure 2A’), suggesting expression of TA in granule cells (Figure 2C’’) and in the endfeets of Bergmann glia (Figure 3A). These latter cells are specialized astroglia and their cell bodies are located in a row within or beneath the PCL. Each glial cell gives rise to several ascending processes (Figure 3B and C) that traverse the ML at a right angle to the surface where they form conical endfeet and come into contact with the pia. The antibody anti-GFAP labels glial cells in the cerebellar cortex (green, Figure 3); TA was localized in the ascending processes and endfeets of Bergmann glia (mTA/GFAP, Figure 3A) and clearly in their cell bodies (Figure 3B–B’). In fact, serial optical sections along Bergmann glia cell body (Figure 3B’, z step 0.3 µm) highlight the presence of the colocalization mask TA/GFAP throughout the cell, indicating the presence of TA in this cell type. To further support this observation we performed a quantitative analysis of the pixel colocalization of TA and the GFAP signals in isolated cell bodies (Figure 3D, left panel). In particular, we provided the values of the *overlap coefficient r* [37–39]: a value of r equal to one indicates complete colocalization while zero indicates no colocalization. To confirm the reliability of the colocalization analysis we reported also the r index of negative CTRs (see methods) represented in Figure 3C (mCb/GFAP panel) Figure 3D). A statistical analysis confirmed a significant difference between the r indexes (TA/GFAP, r = 0.643 ± 0.0198 SEM; Cb/GFAP r = 0.357 ± 0.024 SEM; n = 6 each group; t-test, p < 0.001; Figure 3D left panel).

The granule cells, which are the smallest and most numerous cells in the cerebellum, are densely packed in the IGL (Figure 2C). During the second postnatal week the IGL expands rapidly and continues until the end of the third week, the time when the EGL disappears. The settling of granule cells is directly associated with the development of synapses between granule dendrites and MF terminals in distinctive loci in the IGL, the cerebellar glomeruli. Other neuronal elements are present in the IGL: Golgi cells, Lugaro cells and unipolar brush cells [40]. The soma of the Golgi cell is distinguished from granule cells by its large size, their dendrites arborize mostly in the ML. Golgi cells are GABA-ergic neurons and exert inhibitory action on granule cells in the glomeruli. TA was clearly expressed in both granule cells and Golgi cells (Fig. 2C’-C’’). We also detected TA expression in Lugaro cells which have a typical spindle-shaped morphology and localization (data not shown). We did not investigate TA localization in unipolar brush cells. Finally, we verified the expression of TA also in the bushy astroglia which is localized in the IGL (Figure 3E-F). Of note, a variable expression of TA characterizes also this cell type as highlighted by the different intensity of the two colocalization masks in Figure 3E (high mTA/GFAP) and 3F (low mTA/GFAP). Finally, the r index measured in the glial cell bodies of the EGL (TA/GFAP, r = 0,66 ± 0.0435 SEM; Figure 3D right panel) was significantly different from the value of the negative CTR (Cb/GFAP, r = 0,277± 0.034 SEM; t-test, p <0.001, n = 6 each goup, Figure 3D right panel) represented in Figure 3G. These observations provide evidence that TA is highly localized in cerebellar glial cells.

### *TA localization in the glutamatergic and GABA-ergic synapses of the mouse cerebellum at P14*

**Figure 4:**
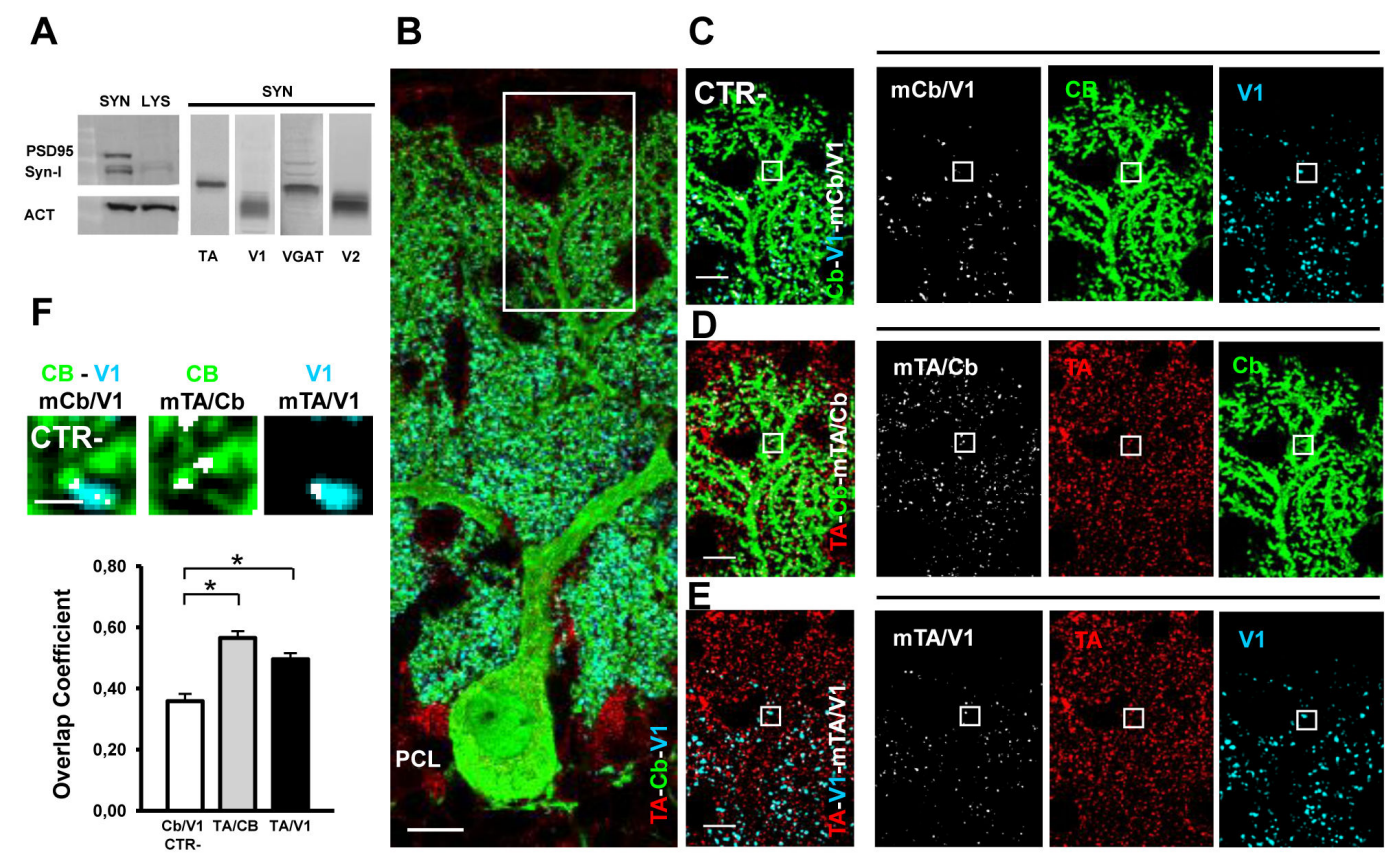
Synaptic localization of TA at the PC–PF synaptic contacts. Western blot of TA, V1, V2, VGAT proteins in cerebellar synaptosomal fractions (SYN) from P14 mice. Synaptosomal preparations are enriched of post synaptic density protein (PSD95) and Synapsin-I (Syn-I) in comparison to equal amount (ACT) of total cerebellar protein extract (LYS). TA is clearly present in synaptosomal enriched preparations. **B**) Cerebellar sagittal section immunostained for Cb (green), V1 (cyan) and TA (red). In the ML, anti-V1 specifically labels PF-terminals contacting PC-spines. **C**–**E**) The high magnification of the inset in B is reproduced in different merge images and by the relative split channels. **C**) Merge image of Cb and V1 staining plus the colocalization mask Cb/V1 (mCb/V1 in white); it represents the negative CTR (CTR-). **D**) Merge image of TA and Cb staining plus the colocalization mask TA/Cb (mTA/Cb in white), highlighting TA expression in PC-spines. **E**) Merge image of TA and V1 staining plus the colocalization mask TA/V1 (m TA/V1 in white), indicating the presence of TA also in this synaptic terminal. **F**) Quantitative colocalization analysis shows the mean overlap coefficients of TA/Cb (gray column) and TA/V1 (black column) significantly different from the negative CTR Cb/V1 (white column). The small insets (white boxes in C–D-E) are high magnifications of a representative PC-spine (green) contacted by a PF synaptic terminal (cyan) with the appropriate colocalization mask (white). * p < 0.05. Data are represented as mean ± SEM. Scale bars in B, 10 µm; in C–D-E, 5 µm; in F, 1 µm.

A detailed characterization of TA localization in the synaptic terminals of the mouse brain was lacking. First we wanted to verify TA presence in protein extracts derived from cerebellar synaptosomal enrichments (Figure 4A). We isolated synaptosomes from cerebella of P14 mice and observed by western blot abundant levels not only of typical synaptic markers (PSD-95 and Synapsin-I) but also of TA, indicating the presence of this protein in the cerebellar synaptic structures.

Next, we characterized TA expression in the main cerebellar synaptic inputs by performing immunofluorescence experiments with antibodies against proteins specifically localized in the synaptic terminals of excitatory and inhibitory neurons; V1 or V2 and VGAT, respectively. The specificity of these antibodies was tested by western blot in cerebellar synaptosomal preparations (Figure 4A). Then, we performed a quantitative analysis of the pixel colocalization of TA and the vesicular transporter signals in isolated set of synaptic contacts by providing the overlap coefficient (r index). Of note, for each set of synapses we reported also the negative CTRs (see experimental procedures).

#### Purkinje cells synaptic inputs and output: glutamatergic and GABA-ergic synapses of the molecular layer and deep cerebellar nuclei

The PCs are the only output of the cerebellar cortex exerting a tonic influence on the DCN [41]. In the ML, PCs receive two main excitatory inputs, the PFs and the CFs, and two important inhibitory inputs from stellate and BCs.

In the ML, the localization of PC excitatory innervation changes during postnatal development [42,43]. Multiple CFs innervate the PC body at early postnatal ages (~P2-P5). In a second stage (~P10-P14) the PC dendritic arborization expands and PFs and multiple CFs innervate these dendrites. A final stage (~P21-adult) involves the maturation of the excitatory synapses on PCs which ends with specific innervation of distinct compartments of the PC dendritic arbor: namely the distal spiny dendritic regions are contacted by many PFs while the proximal dendritic part is contacted by one CF. In addition, each input is characterized by distinct electrophysiological properties [44] and by the complementary expression of the VGluT isoforms [45]; the PFs express V1 while the CFs are V2-positive.

Regarding the PC–PF synapses, we analyzed the upper part of the ML to better define isolated synaptic contacts between Cb-positive PC spines (green, Figure 4B–D) and the V1-positive PF terminals (cyan; Figure 4B,C,E). The colocalization masks in Figure 4D (panel mTA/Cb) and 4E (panel mTA/V1) indicate that TA (red) was localized both at the post- and pre-synaptic sites, respectively. For each synapse we identified the relative negative CTR represented in Figure 4C (panel mCb/V1) between the pre- (V1-positive) and post-synaptic site (Cb-positive). A high magnification of a representative synaptic contact between a single PF and a PC spine is showed in Figure 4F, the relative colocalization masks are also reported (mCb/V1; mTA/Cb; mTA/V1). Accordingly, the quantitative colocalization analysis confirmed TA expression at both presynaptic and postsynaptic level (Figure 4F); of note the r index TA/Cb in PC spines was 0.565 (± 0.022 SEM; n = 156) and in PF terminals the r index TA/V1 was 0.495 (± ; 0.021 SEM; n = 156). Both values were significantly different from the negative control (Cb/V1 r = 0.358 ± 0.024 SEM; n = 156; one-way ANOVA, p < 0.001; post-hoc Holm-Sidak test, p < 0.05 for CTR versus TA/Cb and TA/V1)

**Figure 5:**
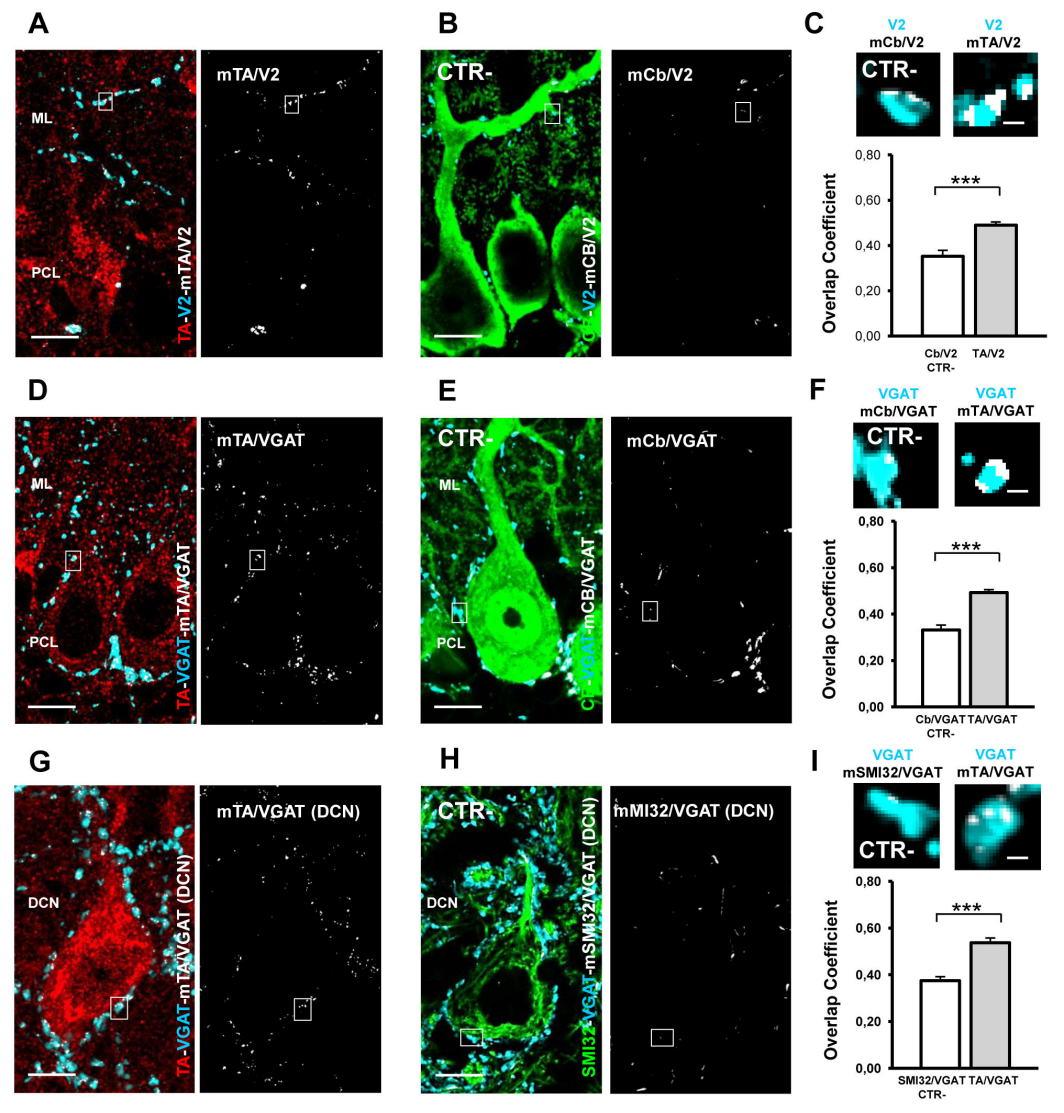
TA is localized in V2-positive CFs, VGAT-positive BC and PC terminals at P14. Merge of TA (red) and V2 (cyan) staining and of the colocalization mask TA/V2 (mTA/V2 white). TA is localized in the V2-positive CF terminals which impinge on the PC proximal dendrite. **B**) The negative CTR (CTR-) is represented by the overlapping signal between the V2-positive CFs (cyan) contacting the Cb-positive PC dendrites (green). The panel on the right is the relative colocalization mask (mCb/V2, white). **C**) Quantitative colocalization analysis of TA in V2-positive terminals. The mean of the TA/V2 overlap coefficients was significantly different from the negative CTR mean value. The insets are high magnification of the white boxes in A and B and show V2 and the relative colocalization masks. **D**) Merge of TA (red) and VGAT (cyan) staining and of the colocalization mask TA/VGAT (mTA/VGAT white) in the PCL and ML. TA is localized in the VGAT-positive terminals of inhibitory neurons contacting PC-dendrites and bodies. **E**) The negative CTR (CTR-) is represented by the overlapping signal between the VGAT-positive terminals (cyan) and the Cb-positive PC bodies (green). VGAT-positive PC collaterals were excluded from the analysis. The panel on the right is the relative colocalization mask (mCb/VGAT, white). **F**) Quantitative colocalization analysis of TA in VGAT-positive terminals (gray column). The mean of the TA/VGAT overlap coefficients was significantly different from negative CTR mean value (white column). The insets are high magnification of the white boxes in D and E showing VGAT and the relative colocalization masks. **G**) Merge of TA (red) and VGAT (cyan) staining and of the colocalization mask TA/VGAT (mTA/VGAT DCN, white) in the DCN region. TA is localized in the VGAT-positive synaptic terminals of PCs which contact DCNs. **H**) The relative negative control was the overlapping signal of VGAT-positive terminals (cyan) impinging on SMI32-positive DCN bodies (green). The right panel is the relative colocalization mask (m SMI32/VGAT DCN, white). **I**) Quantitative colocalization analysis of TA in VGAT-positive terminals (gray column). The mean of the TA/VGAT overlap coefficients was significantly different from negative CTR mean value (white column). The insets are high magnification of the white boxes in G and H and show VGAT and the relative colocalization masks. *** p < 0.001. Data are represented as mean± SEM. Scale bars in A-B, D-E and G-H: 10 µm; in C–F–I: 1 µm.

A similar analysis was performed for the PC–CF synapses. The CF is the terminal arbor of the inferior olive neurons. At P14 the multi CFs regression is almost terminated, therefore most of the PCs bear one CF which carries numerous varicosities. In these glutamatergic terminals (cyan, Figure 5A), TA (red, Figure 5A) was abundantly expressed as shown by the colocalization mask TA/V2 (mTA/V2 in Figure 5A,C) and by the quantitative analysis (Figure 5C). In particular, the r index TA/V2 was 0.49 (± 0.014 SEM; n = 197) and was significantly different from the negative CTR (Cb/V2, r = 0.35 ± 0.027 SEM, n = 83; t-test, p < 0.001; Figure 5B,C).

In the ML, inhibitory neurons are represented by stellate and BCs. Whereas the first are generated since the second postnatal week, the latter start to innervate the PC at around P7 [46]. Thus, we restricted the analysis to the BCs. During development, the axon collaterals of BCs descend along the shaft of the PC stem dendrite and first enwrap the base of the PC soma at P7, making perisomatic synapses. Then BCs axon collaterals reach the PC axon initial segment (AIS) by P9 and after P21 extend in a peculiar structure (pinceau) looking like a nest in the mature stage [46,47]. Therefore at P14 we performed the colocalization analysis on VGAT-positive synaptic contacts around the PC bodies. The colocalization mask in Figure 5D and F (mTA/VGAT) clearly show the presence of TA also in these GABA-ergic terminals (cyan, Figure 5D,F). Accordingly the r index of TA/VGAT (r = 0.493 ± 0.012 SEM; n = 196) was significantly different from the negative CTR (Cb/VGAT, r = 0.33 ± 0.021 SEM; n = 110; t-test, p < 0.001; Figure 5E-F).

Finally we investigated also the GABA-ergic synaptic contacts of PCs which impinge on the DCN, a heterogeneous group of excitatory and inhibitory neurons that receive also collateral projections from the glutamatergic MFs and CFs. Although the precise pattern of synapse development remains to be determined in the DCN, it has been suggested that PC synaptogenesis may start during the perinatal period [41]. Here, we determined TA expression in the PC synaptic inputs which represent the 73% of axon terminals in the DCN neuropil [48]. We observed expression of TA (red, Figure 5G) also in these VGAT-positive synaptic terminals (m TA/VGAT_DCN, Figure 5G,I). The quantification of TA colocalization was indeed significantly higher (TA/VGAT, r = 0,607 ± 0.010 SEM; n = 251) relative to the negative CTR (SMI32/VGAT, r = 0,37 ± 0.027 SEM; n = 158; t-test, p < 0.001; Figure 5H-I). Interestingly, this region of the cerebellum seems to be specifically implicated in dystonia due to its connections to the sensorimotor territory of the striatum via the thalamus [49].

#### Glutamatergic and GABA-ergic synapses of the granular layer: the cerebellar glomeruli

**Figure 6:**
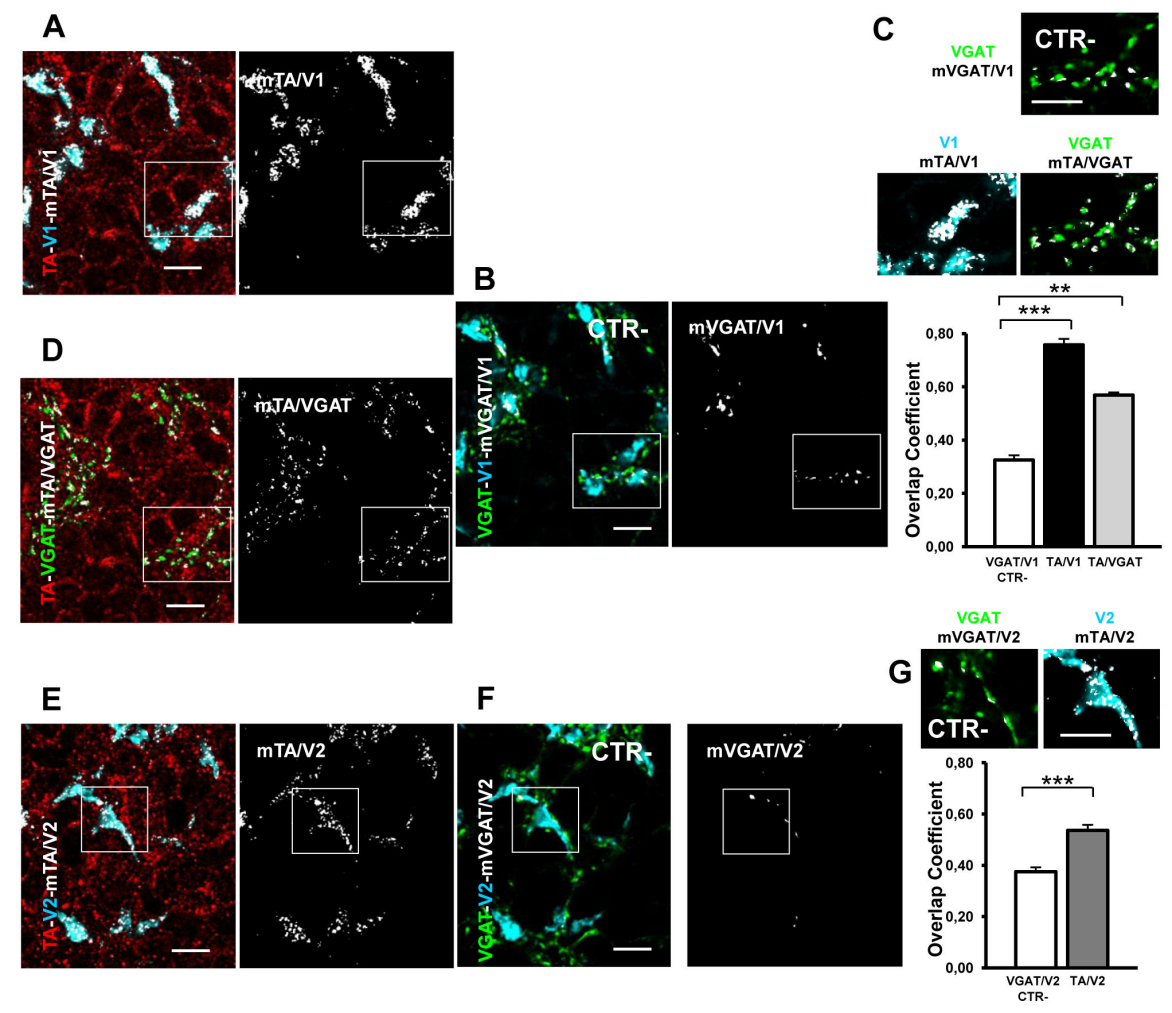
TA expression in glomeruli of the mouse cerebellum at P14. **A**–**G**) In the IGL, V1-positive rosettes (A–B) and V2-positive rosettes (E–F) and VGAT-positive Golgi cell terminals (B–D) contact the granule dendrites in the glomeruli. **A**–**C**) TA colocalization analysis in the V1-positive rosettes. **A**) Merge of TA (red) and V1 (cyan) staining, plus the relative colocalization mask (mTA/V1). **B**) Merge image of V1 and VGAT staining in the glomeruli plus the colocalization mask VGAT/V1 (mVGAT/V1 white, right panel). The overlapping signal represents the negative CTR. **C**) High magnification of the white boxes in A (mTA/V1), B (CTR) and D (mTA/VGAT), respectively. The quantitative colocalization analysis indicates the presence of TA in this excitatory synapse by comparing the mean values of the TA/V1 overlap coefficients (black column) with negative CTR values (white column). **C**–**D**) TA colocalization analysis in the VGAT-positive terminals of Golgi cells in the rosettes (D, mTA/VGAT). The quantitative colocalization analysis in C indicates the presence of TA in this inhibitory synapse by comparing the mean values of the TA/VGAT overlap coefficients (gray column) with negative CTR values (white column). **E**–**G**) TA colocalization analysis in the V2-positive rosettes. **E**) Merge of TA (red) and V2 (cyan) staining, plus the relative colocalization mask (mTA/V2). **F**) Merge image of V2 and VGAT staining in the glomeruli plus the colocalization mask VGAT/V2 (m VGAT/V2 white, right panel). The overlapping signal represents the negative CTR. **G**) high magnification of the white boxes in E (mTA/V2) and F (CTR). Bottom, the quantitative colocalization analysis indicates the presence of TA in this excitatory synapse by comparing the mean values of the TA/V2 overlap coefficients (dark gray column) with negative CTR values (white column). ** p <0.01, *** p < 0.001. Data are presented as mean ± SEM. Scale bars in A–B, and D–F: 10 µm; in C ,G 2,5 µm.

One of the two major glutamatergic inputs to the cerebellum is exerted by the MF. The bulbous MF terminals (called rosettes) form in the granular layer “en passant” synapses with the granule dendrites producing the synaptic complexes called cerebellar glomeruli [42,50]. In particular in a glomerulus each granule dendrite is contacted by one MF and receive also inhibitory synapses by the Golgi cell axons [51]. During development until P15 cerebellar glomeruli go through extensive morphological remodeling, with a progressive increase of synaptic junctions [52] and changes in the excitatory transmission [53]. Although structurally similar, MF rosettes are actually of three distinct types: those that are positive for V1, those positive for V2, and those positive for both [37]. A shown in Figure 6 an abundant expression of TA was evident in V1-rosettes (mTA/V1 in Figure 6A,C) while in the VGAT-positive Golgi terminals (mTA/VGAT, Figure 6 D,C) and V2-positive rosettes (mTA/V2, Figure 6E,G) TA seemed less expressed. The colocalization analysis of TA/V1 and TA/VGAT was performed in the same selected glomeruli to use unique negative CTR (V1/VGAT). The quantitative analysis in Figure 6C show an r index of TA/V1 of 0.758 (± 0.0217 SEM; n = 66 rosettes) and a value of 0.569 (± 0.004 SEM ; n = 66 rosettes) for TA/VGAT. Both experimental groups were significantly different from the negative CTR (V1/VGAT, r = 0.326 ± 0.0174 SEM; n = 66 rosettes; one way-ANOVA p < 0.001 vs TA/V1 and p < 0.01 vs TA/VGAT; Figure 6B,C). Regarding the V2–positive rosettes the r index TA/V2 (r = 0.537 ± 0.021 SEM; n = 42 rosettes; Figure 6G) was also significantly different from the negative CTR (VGAT/V2; r = 0.37 ± 0.022 SEM; n = 42 rosettes; t-test, p < 0.001). These results indicate that the main glutamatergic and GABA-ergic terminals in the IGL express TA.

### *Intensity correlation analysis of TA in the cerebellar synaptic structures*

**Figure 7:**
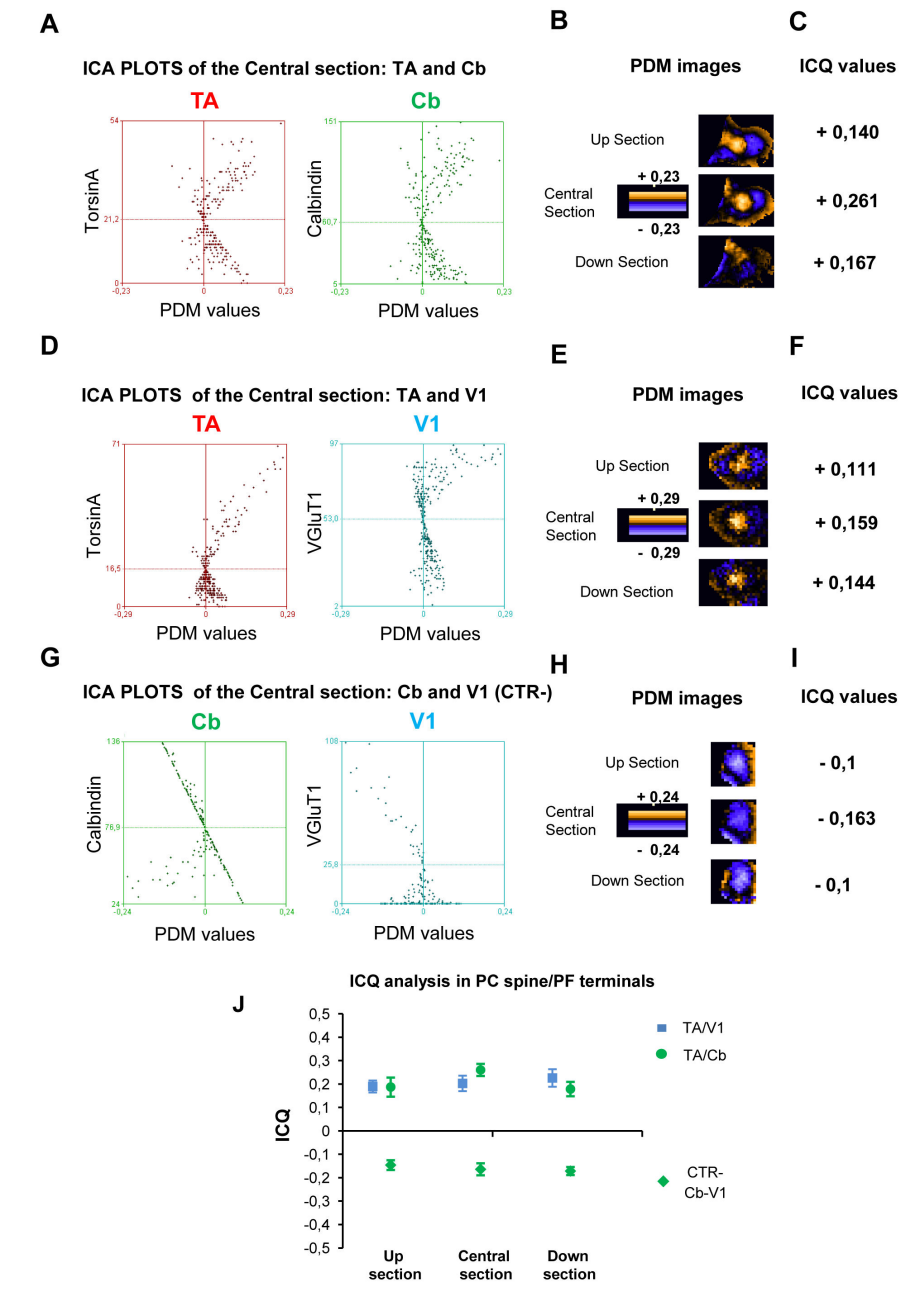
Intensity correlation analysis of TA in PC-spine/PF synapses. **A**–**C**) Representative ICA analysis of TA and Cb at PC-spine in the z-dimension. **A**) ICA plots of TA (left) and Cb (right) staining intensities against their respective PDM values (central section). **B**) Serial sections of PDM images showing positive pixels inside the spine. The image is pseudocolored and a PDM scale bar is shown. **C**) Positive ICQ values (sign test p < 0.001) at each serial section indicate a dependent distribution of TA and Cb in the PC spine. **D**–**F**) Representative ICA analysis of TA and V1 at PF terminals in the z-dimension. **D**) ICA plots of TA (left) and V1 (right) staining intensities against their respective PDM values (central section). **E**) Serial sections of PDM images showing positive pixels inside the PF terminal. **F**) Positive ICQ values (sign test p<0.001) at each serial section indicate a dependent distribution of TA and V1 in the PF. **G**–**I**) Representative ICA analysis of Cb-spine and V1-PF terminal in the z-dimension as control. **G**) ICA plots of Cb (left) and V1 (right) staining intensities against their respective PDM values (central section). **H**) Serial sections of PDM images showing negative pixels inside the spine. **I**) negative ICQ values (sign test p < 0.001) at each serial section indicate a segregated distribution of Cb and V1 in the spine. **J**) Statistical analysis of ICQ values based on multiple contacts. The ICQ values were consistently positive and highly significant not only in the central optical sections but also in the up and down sections for both spines and PF terminals. Accordingly, the ICQ values were consistently and significantly negative along the z-dimension (t-test, p <0.05 relative to 0) in CTR (spine/PF). Of note that the mean values of the three optical sections were not significantly different among them (one-way ANOVA, p < 0.05). Data are represented as mean ± SEM.

It could be argued that the spatial resolution in the z-dimension of light microscopy was not high enough to distinguish between thin structures. Therefore our colocalization analysis conducted on a single optical section could be affected by signals originating from neighboring structures in the z-dimension. To overcome this problem we performed the intensity correlation analysis [38] between TA staining and the synaptic markers along the z-dimension on single TA positive synaptic contacts. In particular, for each synaptic contact we selected three optical serial sections along the z-dimension (0.3 µm step); then for each section we reported the ICA plots, the PDM images and the ICQ values (see Materials and methods). Briefly, ICA plots identify stained pixel pairs that vary in synchrony, randomly or independently within the cell. Plots for dependent or segregated staining patterns generate hourglass figures that are markedly skewed toward positive or negative values, respectively. In the PDM image each pixel is equal to the PDM value at that location (Figure 7B–E-H). ICQ is a statistically testable quotient which provides an overall index of whether the stained protein pairs are associated in a random (ICQ ~ 0), a dependent (0< ICQ <+0.5) or a segregated manner (0> ICQ > -0.5). The normal approximation of the sign test was used to test if these values were significantly different [38].

Representative examples of the ICA are reported in Figure 7 where we analyzed the relative TA intensity signal in a Cb-positive spine (Figure 7A–C) and in a V1-positive PF terminal (Figure 7 D–F). For each synaptic structure the ICA plots (Figure 7A and D) of the central section were strongly skewed toward positive values consistent with highly dependent staining patterns. For simplicity, instead of reporting the ICA plots of the other sections (up and down), we showed the relative PDM images (Figure 7B–E) and the ICQ values (Figure 7C–F) which were significantly positive (sign test, p < 0.001). Furthermore, a statistical analysis of ICQ values based on multiple contacts was performed. As shown in the graph (Figure 7 J) and table 1 the ICQ values were consistently positive and highly significant not only in the central optical sections but also in the up and down sections (n = 5, t-test p < 0.01 versus 0). Of note, the mean values of the three optical sections were not significantly different among them (one-way ANOVA p > 0.05). Finally, we calculated also the relative intensity signal between Cb-positive spines and the V1-positive PF terminals as negative control (Figure 7 G–I). Accordingly, the segregated staining gave hourglass figures with a negative skew (Figure 7G) and the ICQ values along the z-dimension were negative in each optical section (Figure 7I). The analysis was extended to other control structures (Figure 7J and table 1), the ICQ values were consistently and significantly negative along the z-dimension (n = 5, t-test p < 0.05 relative to 0).

**Table 1:** ICQ values of TA in glutamatergic and GABA-ergic contacts.

**synaptic structure**	**ICQ_Up section**	**ICQ_Central section**	**ICQ_Down section**
TA^+^/Cb^+^_spine_	+ 0.19 ± 0.04	+ 0.26 ± 0.03	+ 0.18 ± 0.03
CTR-Cb^+^_spine_ /V1^+^	-0.15 ± 0.02	-0.16 ± 0.03	-0.17 ± 0.02
TA^+^ / V1^+^	+ 0.19 ± 0.03	+ 0.20 ± 0.03	+ 0.23 ± 0.04
CTR-V1^+^/Cb^+^_spine_	-0.14 ± 0.02	-0.13 ± 0.02	-0.15 ± 0.03
TA^+^/V2^+^	+ 0.10 ± 0.03	+ 0.11± 0.02	+ 0,10 ± 0.02
CTR-V2^+^/Cb^+^	-0.14 ± 0.04	-0.19 ± 0.01	-0.17 ± 0.01
TA^+^/VGAT^+^	+ 0.16 ± 0.03	+ 0.15 ± 0.01	+ 0.14 ± 0.01
CTR-VGAT^+^/Cb^+^	-0.18 ± 0.03	-0.18 ± 0.02	-0.15 ± 0.02
TA^+^/VGAT^+^_DCN_	+ 0.11 ± 0.01	+ 0.14 ± 0.02	+ 0.11 ± 0.02
CTR-VGAT^+^_DCN_/SMI32^+^	-0.09 ± 0.01	-0.12 ± 0.03	-0.10 ± 0.02

Data are reported as mean ± SE (n 5 each structure; t-test each section, p<0.05 for all groups; one-way ANOVA among sections of the same structure, p>0.05 for all groups). Abbreviations: ICQ, intensity correlation quotient; TA, TorsinA; Cb, Calbindin; V1-2 vesicular glutamate transporters; VGAT, vesicular GABA transporter; DCN, deep cerebellar nuclei; CTR-, negative control; SMI32, neurofilament H.

The ICA method was applied at the other sets of synaptic terminals previously analyzed. We observed significantly positive ICQ values along the three serial sections confirming TA localization in these synaptic terminals (n = 5 each contact, t-test p < 0.05 relative to 0). Table 1 reports the ICQ values for each set of synaptic structure and their relative controls.

Altogether these analyses provide compelling evidence that TA staining varies in synchrony with each synaptic marker in the z-dimension, consistent with the localization of this protein in the main GABA-ergic and glutamergic synaptic contacts (pre- and post) of cerebellar neurons.

## Discussion

In the present study we examined the expression of TA in different cell populations of the juvenile mouse cerebellum, pointing to its localization in the main GABA-ergic and glutamatergic synaptic terminals. First, we observed that TA was broadly distributed in the cerebellar cortex and DCN without a preferential expression in specific neuronal subtypes. In addition three relevant, novel observations emerged: (i) TA was localized in the spines of PCs; (ii) TA was expressed in the main GABA-ergic and glutamatergic synaptic terminals of the cerebellar cortex; (iii) TA was clearly expressed also in glial cells.

A novel finding of this work, is represented by the characterization of TA expression in the synaptic terminals during postnatal development, at P14, when TA reaches the peak of its expression and cerebellar synaptogenesis occurs [33]. Of note, we conducted a rigorous quantitative analysis by using two different colocalization methods: the first provided the overlap coefficient r for each set of synaptic structures accompanied by the relative negative controls; the second provided the ICQ value for single contacts in the z-dimension. In addition, we supported TA localization in cerebellar synaptic structures (both pre- and post-synaptic) by means of western blot analysis of synaptosomal preparations.

By means of immunofluorescence experiments we observed a staining pattern of TA protein similar to that described previously by immunohistochemistry in adult human and rodent [26,28,30] cerebellum. Besides validating the presence in the DCN, PCs and BC in the ML [26], we could identify the expression of TA in the cell bodies of granule cells, Golgi cells in the granular layer. Our observations are in accordance with quantitative *in situ* hybridization where TA mRNA signal intensity was greatest in regions with high neuronal density such as the IGL of the cerebellum [30]. We noticed that cells belonging to the same neuronal or glial populations (i.e., PC, BC) show a variable expression of TA. In addition, despite TA was not observed in most of the nuclei, we observed a nuclear localization in those cells which expressed high levels of TA.

Several studies on TA have indicated the primary location of TA in the ER lumen where it typically forms oligomeric complexes and likely acts as a chaperone [54]. Here, we detected the presence of a punctuate staining of TA also along dendrites, dendritic spines and axons in particular of PCs. Such distribution is in accordance with the presence of a local ER network which plays critical neuronal function. Interestingly, in dendrites and in spines the ER network allows the local translation of mRNAs encoding signal peptide-containing proteins, thus by passing the somatic ER [55], suggesting a role for TA in synaptic plasticity events [56]. However, besides its localization in the ER, TA has been detected in the cytosol and in neurite processes [11,12] as part of a protein complex which included cytoskeletal elements such as vimentin, actin, tubulin [9], and the motor protein, kinesin light chain 1 [13]. Interaction with cytoplasmic partners suggests that there may be a form of TA which has access to the cytoplasm or that, through binding to other ER-associated proteins which extend out of the ER, TA may take part in a protein complex that spans from the lumen of the ER to the cytoplasm. By interacting with the cytoskeletal network TA may contribute to control neurite outgrowth and could be involved in maintaining cell polarity [23]. Accordingly, its developmental regulation suggests that TA may play a role in postnatal maturational events in the CNS [30], such as dendritic and axonal arborization. Here, we observed that TA is highly expressed in different cerebellar neurons, such as PCs and DCN. A major novel finding of the present work is the localization of TA not only in the dendritic arbor but also in the spines of developing PCs. Of note, it has been recently observed that a reduced expression of TA selectively in PCs induced alterations of PC development resulting in shortened primary dendrites and decreased spine density [57]. Furthermore, most of the output fibers of the cerebellum originate in the DCN. Interestingly, DYT1 heterozygous knock-in mice, which carry a mutant TA, exhibited subtle WM abnormalities in cerebello-thalamo-cortical motor pathways similar to those identified in human gene carriers [6].

Moreover, TA might be involved in the correct spatial distribution of neuron output. Accordingly, one of the best example of neuronal class-specific innervation patterns is found in cerebellum. In particular, PCs which reside in the translobular plane of the cerebellar cortex receive two excitatory and two inhibitory synaptic inputs impinging on well defined territories of the dendritic arbor [50]. Importantly, in the ML the Bergmann glia cells are positioned to interact with multiple neuronal components and contribute, at different developmental stages, to multiple aspects of cerebellar circuit assembly, including spinogenesis and axon arborization [47,58]. Here, we demonstrated that TA is also expressed by this cell type where it might play a role in maturation. Bergmann glia are highly polarized astrocytes, whose radial fibers dominate the cerebellar cortex [50,59,60]. During postnatal cerebellar development, the apical Bergmann glia fibers form the earliest radial structures across the cerebellar cortex and develop characteristic endfeet at pia level. Here, we observed a strong presence of TA not only in the Bergmann glia cell bodies and radial fibers (ML) but also in the astroglia which populate the IGL. Of note, this is the first time that TA is clearly detected in glial cells although its expression is variable. TA seems to be upregulated in glia cells following stress [61], suggesting that in physiological conditions its expression level is very low. In adult human brain, where TA expression is more heterogeneous relative to rodent brain [62,63], TA was almost undetectable in glia cells [28].

In recent years, different functional roles have been attributed to TA including the involvement in the secretory pathway [16,18], synaptic vesicle turnover [13] and release [19]. Both *in vitro* and *in vivo* studies have suggested that TA may act as a chaperone, not only at the ER but also at the synapse, by mediating the transport, assembly and disassembly of the molecular complex and consequently affecting SV turnover and neurotransmitter release [20–23]. Ultrastructural studies of primate and human striatum have shown that TA immunostaining is associated with small clear vesicles within axons and presynaptic terminals, and it is also found enriched in synaptosomal fractions [24]. Here we provided for the first time a detailed analysis of TA localization in the main synaptic terminals of juvenile cerebellum in correlation to extensive synaptogenesis. We observed a quite homogenous distribution in both glutamatergic and GABA-ergic synapses. As already mentioned a crucial aspect of synaptogenesis is the detection of the target. The selection of a specific synaptic input depends on different events, such as the guidance of axons to their appropriate targets, the release of diffusible factors, and signaling mediated by trans-synaptic adhesion complexes [64,65]. Curiously, a novel class of secreted synaptic organizers has been identified as crucial player for synapse formation and maintenance [66]. TA interacts with sarcoglycans [67] which are components of the dystrophin-glycoprotein complex involved in GABA-ergic synapse development [68]. In addition, synaptic activity is thought to mediate competition between convergent inputs, leading to the strengthening or elimination of an immature synapse [69]. For example the transition from multiple to single innervation of PCs by CFs is one of the best systems to investigate activity-dependent synaptic competition that underlies the refinement of synaptic circuits in the central nervous system [43,70]. Recent evidence has provided important information on the activity-dependent mechanisms that regulate the refinement of synaptic circuits and determine connectional specificity in the cerebellum [33]. If TA, by regulating SV transport and turnover, plays a role in the molecular and/or activity-dependent mechanisms that control the spatial specificity of synaptogenesis has to be investigated.

## Conclusions

Despite the function of TA is still not completely understood, the present observations provide the basis for improving our knowledge of TA role(s) in cerebellar development. Moreover, TA is directly involved in a primary form of dystonia, DYT1 dystonia. A mutant form of TA seems to be responsible of subtle developmental alterations which in turn cause neuronal functional abnormalities leading to dystonic movements [71]. Both the age window of susceptibility and the phenotype of DYT1 seem to be correlated to the time course of synaptic maturation in the striatum and/or cerebellum. Therefore, our obervations might have potentially relevant implications for the pathophysiology of DYT1 dystonia.
